# Evolução do Tratamento e o Impacto dos Fatores Preditores Pré-Cirúrgicos nos Desfechos de Pacientes com Doença Cardíaca Congênita

**DOI:** 10.36660/abc.20220020

**Published:** 2022-02-14

**Authors:** Maurice Zanini

**Affiliations:** 1 Universidade Federal do Rio Grande do Sul Porto Alegre RS Brasil Universidade Federal do Rio Grande do Sul,Porto Alegre, RS – Brasil

**Keywords:** Cardiopatias Congênitas/cirurgia, Período Pré-Operatório, Medição de Risco, Estudos Retrospectivos, Fatores de Risco, Sobrevivência

Dentre as malformações, a doença cardíaca congênita (DCC) é a classe mais comum. São malformações importantes que podem comprometer tanto a sobrevida como a qualidade de vida do paciente. Embora haja uma ligeira variação entre muitos estudos de base populacional, a DCC ocorre em aproximadamente 1% dos nascidos vivos (os dados variam de 4 a 10 em cada 1000 nascidos vivos), com prevalência semelhante em todo o mundo. É também a principal causa de mortalidade desde o nascimento, sua incidência é considerada alta diante de sua gravidade.^1 - 4^

A DCC crítica costuma ser letal na ausência de tratamento. Desde o primeiro reparo com uso de circulação extracorpórea em 1953, o diagnóstico preciso e o tratamento eficaz tornaram-se factíveis, mesmo tratando-se de lesões cardíacas congênitas mais complexas. As terapias cirúrgicas eficazes aumentaram a expectativa de vida.^1^

A taxa de sobrevivência em crianças com cardiopatia congênita aumentou substancialmente desde a década de 1980, segundo estudo sueco. Atualmente, mais de 97% das crianças com DCC podem atingir a idade adulta.^5^

Apesar da melhora na sobrevida desses pacientes nas últimas décadas, seu manejo ainda é complexo devido à fisiologia cardíaca e pulmonar que são interdependentes. Complicações pulmonares de DCC podem ser estruturais, em decorrência de compressão ou perturbação das forças de Starling. Em certos tipos de DCC, essas alterações estruturais levam a danos na membrana alveolar-capilar e edema pulmonar. Por sua vez, tais danos resultam em pulmões com baixa complacência e com um padrão de função restritivo que pode se deteriorar e evoluir para hipoxemia. Sob tais circunstâncias, a capacidade do coração de aumentar o fluxo sanguíneo sistêmico e/ou pulmonar é frequentemente limitado. A pressão parcial de oxigênio arterial pode ser diminuída por lesões de *shunt* e a distribuição de oxigênio não pode atender às necessidades dos tecidos. Frequentemente, o distúrbio circulatório também exerce pressão sobre o próprio sistema respiratório. Patologias em ambos os sistemas constantemente coexistem e impactam uns aos outros, tornando o diagnóstico e o manejo dos pacientes mais desafiadores.^6^

Ainda nesse contexto, temos as cardiopatias congênitas que podem ser classificadas em cianóticas e acianóticas. As DCC acianóticas ainda são subclassificadas em lesões de *shunt* e lesões obstrutivas.^7^ A cianose central afeta uma parcela pequena de todos os recém-nascidos e geralmente aponta para um distúrbio subjacente grave que pode necessitar de tratamento de emergência.^8^

No Brasil, os achados observacionais apontaram que o perfil dos pacientes com cardiopatias congênitas foi de lactentes, pré-escolares e escolares, sem predomínio de gênero. Observou-se maior prevalência das cardiopatias congênitas acianóticas. Por outro lado, a maioria do número de óbitos estava entre as cardiopatias cianóticas.^9^

Apesar das melhorias significativas na sobrevida precoce após cirurgia cardíaca congênita (CCC), os pacientes ainda têm morbidade e mortalidade significativas a curto e longo prazo. Para desenvolver estratégias mais individualizadas, é necessário aprofundar nosso conhecimento dos fatores preditores que colocam um paciente em risco após CCC. Dados sugerem que fatores preditores não tradicionalmente incorporados aos modelos de risco podem ter um impacto importante nos desfechos. Segundo Pasquali et al.,^10^ esses fatores explicam uma proporção relativamente pequena de variação na mortalidade total. Esta variação sem explicação destaca a necessidade de explorar novos fatores preditores em pacientes que requerem CCC.

Nesta edição do Arquivos Brasileiros de Cardiologia, Inoue et al.,^11^ apresentam informações sobre fatores preditores, como o impacto da capacidade funcional pré-operatória nos resultados pós-operatórios de CCC. Trata-se de estudo observacional que avaliou a associação do condicionamento pré-operatório de crianças e adolescentes com cardiopatias, através do teste de caminhada de 6 minutos (TC6) e variabilidade da frequência cardíaca (VFC), com a ocorrência de choque cardiogênico, choque séptico e morte no período pós-operatório. Os achados sugerem que a dessaturação de oxigênio após a aplicação do TC6 no pré-operatório parece ser um preditor independente de prognóstico. Entretanto, a distância percorrida no TC6 e as variáveis de VFC não apresentaram a mesma associação.

É importante destacar que os valores de saturação periférica de oxigênio (SpO2) identificada como preditor independente de prognóstico são uma medida específica após esforço ao finalizar o TC6. A SpO2 de repouso não apresentou associação significativa com desfechos nos pós-operatórios. Estudos que reportam a SpO2 como um possível preditor de eventos adversos nesses pacientes apresentam a medida do SpO2 em repouso.^12 , 13^ O diferencial que identificamos no presente trabalho de Inoue et al.,^11^ é o desempenho na SpO2 no momento da recuperação ao TC6. Essa variável pode vir a ser uma nova medida relevante na avaliação dessa população estudada.

Na população pediátrica, ainda são escassos estudos sobre a avaliação de risco de mortalidade no seguimento pós-cirúrgico, considerando dados pré-operatórios. Através da Inteligência Artificial, pesquisadores desenvolveram e validaram um modelo de predição de risco de morte pré-operatório em pacientes com DCC submetidos à cirurgia. As variáveis preditoras mais relevantes incluíram: saturação arterial de oxigênio, admissão anterior à UTI, grupo de diagnóstico, altura do paciente, síndrome do coração esquerdo hipoplásico, massa corporal e atresia pulmonar. Essas variáveis preditoras combinadas representam 67,8% da importância para o risco de mortalidade no algoritmo Random Forest. Segundo os autores, um dos maiores desafios no desenvolvimento de preditores de morte relacionada à CCC é a heterogeneidade de anomalias cardíacas congênitas. Ao contrário da cirurgia cardíaca em adultos (onde há um número limitado de procedimentos cirúrgicos e um número muito grande de pacientes submetidos a tais procedimentos), o oposto se aplica à cirurgia cardíaca pediátrica. Nesta última, existem diversas técnicas e pequeno número de pacientes submetidos a cada tipo de procedimento. Assim, para construir um modelo preditivo de risco útil, a experiência de locais com muitos pacientes deveria ser analisada.^13^

Por fim, podemos considerar que abordagens inovadoras para procedimentos, gerenciamento de pacientes e pesquisa clínica impulsionaram o campo da medicina cardiovascular pediátrica desde o seu início. A evolução no diagnóstico e o tratamento dos defeitos cardiovasculares anatômicos em um ambiente hospitalar apresentam potencial para o desenvolvimento de novos modelos de atendimento. Esses modelos serão centrados no paciente e cada vez mais personalizados, com abordagens mais assertivas, dado o perfil de risco individual. Evitando assim eventos adversos, otimizando a sobrevida, a funcionalidade e a qualidade de vida dos pacientes.^1^
