# Supplementary material for: Evolução do Tratamento e o Impacto dos Fatores Preditores Pré-Cirúrgicos nos Desfechos de Pacientes com Doença Cardíaca Congênita
Source: Arq Bras Cardiol. 2022 Feb 14;118(2):420–1. [Article in Portuguese] doi: 10.36660/abc.20220020 (PMC8856684; doi:10.36660/abc.20220020)
Supplement: Translation

# Translation: Treatment Evolution and the Impact of Pre-Surgical Predictors on Outcomes of Patients with Congenital Heart Disease

**Authors:** Maurice Zanini

**Affiliations:** 1 Brazil Universidade Federal do Rio Grande do Sul, Porto Alegre, RS – Brazil

**Keywords:** Heart Defects,Congenital/surgery, Pre-Operative Period, Risk Assessment, Retrospective Studies, Risk Factors, Survival

Congenital heart disease (CHD) is the most common class among malformations. These are important malformations that can compromise the patient’s survival and quality of life. Although there is slight variation among many population-based studies, CHD occurs in approximately 1% of live births (data range from 4 to 10 per 1000 live births), with a similar prevalence worldwide. It is also the main cause of mortality from birth, and its incidence is considered high given its severity.^1 - 4^

Critical CHD is often lethal in the absence of treatment. Since the first repair using cardiopulmonary bypass in 1953, accurate diagnosis and effective treatment have become feasible, even in the case of more complex congenital heart lesions. Effective surgical therapies have increased life expectancy.^1^

According to a Swedish study, the survival rate in children with congenital heart disease has increased substantially since the 1980s. Currently, more than 97% of children with CHD can reach adulthood.^5^

Despite the improvement in the survival of these patients in recent decades, their management is still complex due to the interdependent cardiac and pulmonary physiology. Pulmonary complications of CHD can be structural due to compression or disruption of Starling forces. In certain types of CHD, these structural changes damage the alveolar-capillary membrane and pulmonary edema. In turn, such damage results in low-compliance lungs and a restrictive pattern of function that can deteriorate and progress to hypoxemia. Under such circumstances, the heart’s ability to increase systemic and/or pulmonary blood flow is often limited. Shunt injuries can decrease partial pressure in arterial oxygen, and oxygen delivery cannot meet tissue needs. Often, the circulatory disturbance also puts pressure on the respiratory system itself. Pathologies in both systems often coexist and impact each other, making diagnosis and patient management more challenging.^6^

Still, in this context, we have congenital heart diseases that can be classified as cyanotic and acyanotic. Acyanotic CHDs are still subclassified into shunt lesions and obstructive lesions.^7^ Central cyanosis affects a small proportion of all newborns and usually points to a serious underlying disorder that may require emergency treatment.^8^

In Brazil, observational findings showed that the profile of patients with congenital heart disease was infants, preschoolers and schoolchildren, with no predominance of gender. A higher prevalence of acyanotic congenital heart disease was observed. On the other hand, most deaths were among cyanotic heart diseases.^9^

Despite significant improvements in early survival after congenital heart surgery (CHS), patients still have significant short- and long-term morbidity and mortality. To develop more individualized strategies, we must deepen our knowledge of the predictors that put patients at risk after CHS. Data suggest that predictors not traditionally incorporated into risk models can have an important impact on outcomes. According to Pasquali et al.,^10^ these factors explain a relatively small proportion of variation in total mortality. This unexplained variation highlights the need to explore new predictors in patients requiring CHS.

In this issue of *Arquivos Brasileiros de Cardiologia* , Inoue et al.^11^ present information on predictive factors, such as the impact of preoperative functional capacity on postoperative CHS results. This observational study evaluated the association of preoperative conditioning in children and adolescents with heart disease, through the 6-minute walk test (6MWT) and heart rate variability (HRV), with the occurrence of cardiogenic shock septic and death in the postoperative period. The findings suggest that oxygen desaturation after applying the 6MWT preoperatively appears to be an independent predictor of prognosis. However, the distance covered in the 6MWT and the HRV variables did not show the same association.

It is important to highlight that peripheral oxygen saturation (SpO2) values identified as an independent predictor of prognosis are a specific measure after exertion at the end of the 6MWT. Resting SpO2 was not significantly associated with postoperative outcomes. Studies that report SpO2 as a possible predictor of adverse events in these patients have measured SpO2 at rest.^12 , 13^ The difference that we identified in the present study by Inoue et al.^11^ is the performance in SpO2 at the time of recovery to the 6MWT. This variable may prove to be a new relevant measure in evaluating this population studied.

There are still few studies on the assessment of mortality risk in the postoperative follow-up in the pediatric population, considering preoperative data. Using Artificial Intelligence, researchers developed and validated a model for predicting the risk of preoperative death in CHD patients undergoing surgery. The most relevant predictor variables included: arterial oxygen saturation, previous ICU admission, diagnostic group, patient height, hypoplastic left heart syndrome, body mass, and pulmonary atresia. These combined predictor variables account for 67.8% of the importance of mortality risk in the Random Forest algorithm. According to the authors, one of the biggest challenges in developing predictors of CHS-related death is the heterogeneity of congenital cardiac anomalies. Unlike adult cardiac surgery (where there are a limited number of surgical procedures and a very large number of patients undergoing such procedures), the opposite applies to pediatric cardiac surgery. There are several techniques and a small number of patients undergoing each type of procedure in the latter. Thus, to build a useful predictive risk model, the experience of sites with many patients should be analyzed.^13^

Finally, we can consider that innovative approaches to procedures, patient management and clinical research have driven the field of pediatric cardiovascular medicine since its inception. The evolution in the diagnosis and treatment of anatomical cardiovascular defects in a hospital environment presents the potential for developing new models of care. Given the individual risk profile, these models will be patient-centered and increasingly personalized, with more assertive approaches, thus avoiding adverse events optimizing patient survival, functionality and quality of life.^1^
